# Current View on the Mechanisms of Alcohol-Mediated Toxicity

**DOI:** 10.3390/ijms22189686

**Published:** 2021-09-07

**Authors:** Anna Birková, Beáta Hubková, Beáta Čižmárová, Beáta Bolerázska

**Affiliations:** 1 Department of Medical and Clinical Biochemistry, Faculty of Medicine, Pavol Jozef Šafárik University in Košice, 04011 Kosice, Slovakia; 2 1st Department of Stomatology, Faculty of Medicine, Pavol Jozef Šafárik University in Košice, 04011 Kosice, Slovakia

**Keywords:** ethanol, acetaldehyde, metabolization, oxidative stress, toxicity

## Abstract

Alcohol is a psychoactive substance that is widely used and, unfortunately, often abused. In addition to acute effects such as intoxication, it may cause many chronic pathological conditions. Some of the effects are very well described and explained, but there are still gaps in the explanation of empirically co-founded dysfunction in many alcohol-related conditions. This work focuses on reviewing actual knowledge about the toxic effects of ethanol and its degradation products.

## 1. Introduction

**Figure 1:**
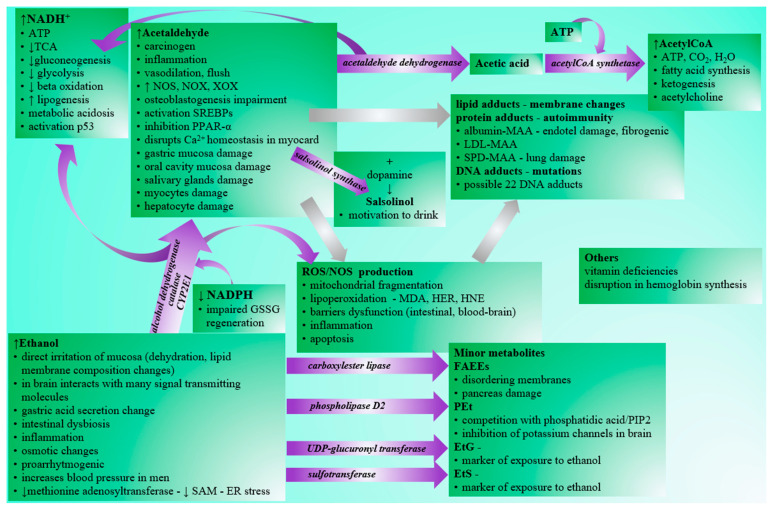
Pathological effects of ethanol and its metabolites. Legend: NADH—reduced nicotinamide adenine dinucleotide, ATP—adenosine triphosphate, TCA—tricarboxylic acid cycle, SAM-S—adenosyl methionine, ER—endoplasmic reticulum, NOS—NO synthase, NOX—NADPH oxidase, XOX—xanthine oxidase, GSSG—oxidized glutathione, SREBP—sterol regulatory element binding proteins, PPAR—alpha-peroxisome proliferator-activated receptor alpha, MDA—malondialdehyde, HER—hydroxyethyl radical, HNE—hydroxynonenal, MAA—acetaldehyde-malondialdehyde adduct, LDL—low-density lipoprotein, SPD—surfactant protein D, DNA—deoxyribonucleic acid, PIP2—phosphatidylinositol diphosphate, FAEE—fatty acid ethyl ester, PEt—phosphatidyl ethanol, EtG—ethyl glucuronide, EtS—ethyl sulfate.

Although the negative effects of excessive alcohol consumption are generally known in the human population (Figure 1), the drinking of alcoholic beverages is prevalent in society. According to WHO, alcohol abuse contributes to three million deaths per year globally and millions of people’s disabilities and poor health. It is alarming that alcohol abuse is the cause of up to 10% of all deaths in the age group 15–49 years [1]. Acute intoxication is often the limit for ending ongoing consumption. In addition to acute intoxications, which often require urgent care attention, especially in young people, the danger of alcohol consumption lies primarily in alcohol addiction, leading to chronic abuse and organ damage. Affected organs/systems include the liver [2], the cardiovascular system [3], the endocrine system [4], the metabolism of basic nutrients [5], the neural system [6,7], or the gastrointestinal tract [8]. Although the mentioned organs/systems perform different functions, the distribution and metabolization of ethanol in organs/systems are variable. At the same time, there are also interrelationships in ethanol-related tissue damage, e.g., gastrointestinal or hepatic impairment may be associated with dysregulation of the immune system and vice versa [9]. There is also a relationship between chronic alcohol abuse and many types of cancer [10]. 

## 2. Alcohol Intake

The most common route of alcohol ingestion is oral. Ethanol uptake by inhalation or other routes is less common. Under some circumstances, ethanol can be created by one’s own body. The bacteria’s natural fermentation of saccharides in the gastrointestinal tract may lead to the production of trace amounts of endogenous ethanol. Dysmicrobia caused by *Candida albicans* or *Saccharomyces species* may promote ethanol production in the gut, as they produce acetaldehyde as the end product of anaerobic metabolism of pyruvate via the action of pyruvate decarboxylase. Accumulation of the acetaldehyde produced may lead to the synthesis of endogenous ethanol by a reversible reaction via bacterial alcohol dehydrogenase. This autogeneration of ethanol is not satisfactory to provide significant concentrations in peripheral venous blood from a forensic point of view [11]. However, nowadays, studies also confirm the effect of ethanol autoproduction on several pathological conditions, such as the increased intestinal permeability associated with liver steatosis [12] and non-alcoholic fatty liver disease [13,14]. In addition to the previously mentioned bacteria, also Gram-negative *Klebsiella pneumoniae* [15], *Escherichia coli* [16], and obligate anaerobes from *Clostridia* class [14] may colonize the gut of endogenous ethanol producers with possible pathological consequences. The production of endogenous ethanol and its metabolite acetaldehyde shows seasonal variation, and its ratio is important in body balance control during adaptation to cold [17].

Orally ingested alcohol is rapidly absorbed. The absorption of ethanol from the gut is dependent on several factors. These may be the time of the day, hydration state, the dosage and concentration of ingested ethanol, or the fed/fasting state of the drinking individual [18]. Ethanol passes freely through the biological membranes according to the concentration gradient and quickly reaches equilibrium with the plasma content. First-pass metabolization is present by the stomach and liver [19]. Ethanol is transported freely in plasma, without plasma protein transporter required. The transfer into the tissues can be affected by the relative water content of the tissues or cells, so that the distribution of ethanol may vary between tissues [20]. Therefore, the volume of ethanol distribution is lower during dehydration and in women or the elderly due to lower muscle mass [18]. A minor fraction of ingested ethanol undergoes biotransformation prior to urinary excretion via glucuronidation to form ethyl glucuronide EtG (0.6–1.5%) or via sulfation to form ethyl sulfate EtS (0.1%) [19,21]. Both glucuronidation and the sulfation of ethanol are possible in the lungs and liver, and the median time to appearance of ethyl glucuronide and ethyl sulfate is approximately 20 h [22]. In trace amounts, ethyl glucuronide accumulates in the hair and is currently one of the most reliable biomarkers of long-term alcohol exposure [23,24]. Most ethanol is oxidized via several possible enzymatic pathways, and the liver is the major organ (but not the only one) for its metabolization.

## 3. Metabolization of Ethanol

### 3.1. Oxidation of Ethanol

#### 3.1.1. Conversion of Ethanol to Ethanal

A few pathways of enzymatic oxidation of ethanol to ethanal in humans are covered below.

Alcohol dehydrogenase (ADH) is a zinc-dependent dimeric enzyme with generally low Km (0.05–0.1 g/L) for ethanol [18] located mainly in the cytosol, which converts several types of alcohols to aldehydes. The conversion of ethanol to acetaldehyde proceeds according to the reaction:



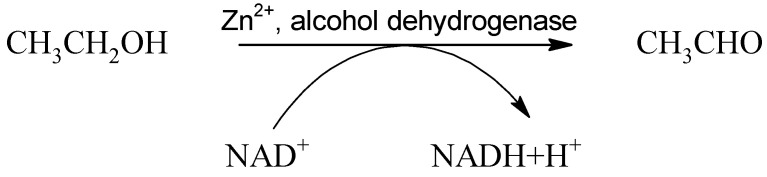

(1)


The resulting NADH is reoxidized in the electron transport chain and thus represents a source of ATP. NADH reoxidation is a limiting process of ethanol metabolism, and high electron transport chain activity is vital in influencing the onset of symptoms of acute poisoning. The enzyme saturates very quickly, and ethanol oxidation by ADH is considered the main pathway of metabolization and is active mainly in light or moderate drinking. The enzyme displays polymorphisms that are responsible for racial and ethnic variations in ethanol pharmacokinetics. There are five classes of the ADH enzyme, which can exist as homo- or heterodimer, consisting of various subunits. Class I of ADH subunits α, β, and γ are encoded by genes *ADH1A (ADH1), ADH1B (ADH2),* and *ADH1C (ADH3),* respectively. ADH1-3 isoenzymes share ≈93% sequence identity but differ in their substrate specificity and developmental expression. This class I is responsible for approximately 70% of the total ethanol oxidation capacity. Other classes of isoforms consist of class II (ADH4, dimer of subunit π), class III (ADH5, dimer of subunit χ), class IV (ADH7, subunits μ or σ), and class V (ADH6) [25,26]. The class I variant ADH1A is active mainly in infants and plays only a minor role in the ethanol oxidation in adults [27]. Opposites are variants of ADH1B or ADH1C, which are the major isoforms responsible for the oxidative pathway of ethanol [28,29]. Based on the Human Protein Atlas, tissue expression, cell compartmentalization, possible substrates, and other specific notes on ADH isoforms are listed in Table 1 [27,28,29,30,31,32,33,34,35,36,37,38,39,40,41,42,43,44,45,46].

Information on the variable expression of ADH points to an essential role of organs other than the liver in ethanol metabolism and possible toxic effects in non-liver tissues. Cells within the gastrointestinal tract expressing various forms of ADH are vital for the first-pass effect of ethanol [47]. Lungs are an important organ for the excretion of unchanged ethanol and the ethanol’s biotransformation and oxidation [48]. 

The structure of ADH and the reaction mechanism are currently thought to be well described and understood [25,49], but a new report on the requirement of ATP for the ADHA1 action is not yet included in textbooks [50]. In addition, inhibition of ADH activity may lead to increased ethanol metabolism via other pathways. An example is aspirin and salicylate, with the variable extent of inhibition of multiple forms of ADH isoenzymes, which may be competitive or non-competitive [51], so the concomitant use of ethanol with widely used drugs with this effect may aggravate the formation of other potentially harmful metabolic intermediates and byproducts.

Cytochrome P450 2E1 (CYP2E1) is a membrane-bound enzymatic system with a higher Km (0.5–0.8 g/L) for ethanol compared to ADH [18], which is present exclusively in the endoplasmic reticulum and mitochondria of hepatocytes [52]. It is inducible and plays an essential role in ethanol metabolism, especially in heavy drinkers. Low and high concentrations of ethanol induce its activity but via distinct pathways. Low concentrations lead to stabilization of the CYP2E1 apoprotein, and high concentrations induce *CYP2E1* via activation of de novo transcription. This oxidation system is also involved in the biotransformation of a wide range of xenobiotics, many endogenous substances [18], and has a unique role in processing carcinogens from the environment [53]. Thus, the oxidation of ethanol by CYP2E1 hemoprotein can have high variability and proceeds according to the following interaction:



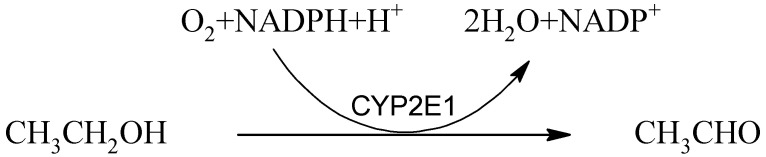

(2)


Important by-products of this reaction are reactive oxygen species [54], which, together with disruption of the glutathione regeneration due to the consumption of NADPH + H^+^, contribute to oxidative stress, thus leading to tissue damage. 

Peroxisomal catalase is highly expressed in hepatocytes but is also distributed in other types of cells, e.g., glandular cells of the duodenum, intestine, adrenal glands, and prostate, tubular cells in kidneys, and macrophages of lungs [55]. Catalase is an antioxidant enzyme primarily catalyzing the decomposition of hydrogen peroxide into oxygen and water. The reaction of ethanol oxidation by catalase is as follows:



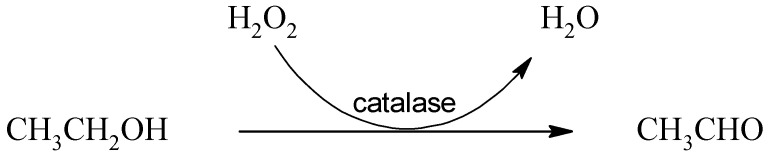

(3)


Although this enzyme is reported to be involved in ethanol metabolization, some authors claim that the reaction is improbable to occur under in vivo conditions due to the lack of hydrogen peroxide necessary for the reaction [18]. Other authors highlight the significant role of catalase in the brain, in which this enzyme may be responsible for the conversion of 60% of ethanol to acetaldehyde. Oxidation by CYP2E1 also occurs in the brain, and the alcohol dehydrogenase action is minor, if any [56,57]. This claim is supported by the fact that the brain produces acetaldehyde from ethanol even under conditions with pyrazole-inhibited alcohol dehydrogenase [58]. Under conditions with high activity of aldehyde oxidase and xanthine oxidase, which are important producers of hydrogen peroxide, the oxidation of ethanol by the action of catalase in the liver also increases [59,60].

#### 3.1.2. Conversion of Ethanal to Acetate and Fate of Acetate

Acetaldehyde produced by the oxidation of ethanol is classified as a category 2 carcinogen according to the Annex VI regulation (EC) 1272/2008. However, physiologically, it is present in low background concentrations, and its toxicity is limited to conditions with higher concentrations [61]. Acetaldehyde is found in some alcoholic beverages as a by-product of alcoholic fermentation [62] but can also enter the body through cosmetics, tobacco smoking, or the consumption of certain foods, such as dairy products, various fruits, or vegetables [63]. In the body, acetaldehyde is further oxidized to acetic acid by the enzyme aldehyde dehydrogenase (ALDH):



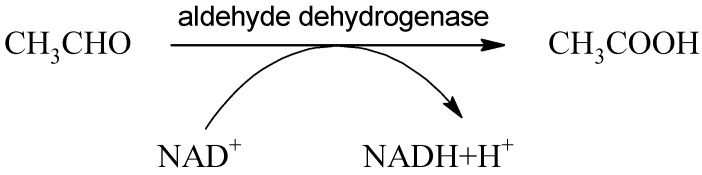

(4)


Acetaldehyde cannot cross the blood–brain barrier and is distributed in two compartments: the peripheral and central nervous systems [64]. Aldehyde dehydrogenase is a superfamily of 19 isozymes catalyzing the oxidation of various aldehydes into less toxic acids. Acetaldehyde is the substrate of the following four ALDH members: cytosolic ALDH1A1 (ALDH1, Km 50–100 μmol/L), mitochondrial ALDH1B1 (ALDH5, Km 30 μmol/L), mitochondrial ALDH2 (Km < 5 μmol/L) [65], and cytosolic and plasma membrane-bounded ALDH3A1 (ALDH3), which can process acetaldehyde in the presence of some natural compounds such as safrole [66] present, for example, in cinnamon and black pepper, or a sulforaphane compound present in cruciferous vegetables [67]. Ethanol as the precursor of acetaldehyde can stimulate ALDH3 activity [68]. Hepatic ALDH2 is responsible for approximately 50% of the circulating acetaldehyde clearance after alcohol ingestion [69], and acetaldehyde represents the primary substrate for ALDH2 [70]. All three forms are expressed in different cell types of various tissues [71,72,73]. ALDH2 shows many polymorphisms. The point mutation of ALDH2 present in more than 40% of the East Asian population is responsible for sensitivity to alcohol consumption [74] and very high risk of squamous cell carcinoma of upper aerodigestive tract due to the accumulation of toxic acetaldehyde [75]. The alteration in NAD binding leads to a loss of catalytic activity and the resulting accumulation of toxic ethanal. An example is the variant of ALDH 2 caused by the single nucleotide polymorphism E487K associated with cardiovascular diseases, alcohol intolerance, or Alzheimer’s disease, which is responsible for a 200-fold increase in K_M_ for NAD [70]. This polymorphism exhibits significant instability and deviation compared to the wild type, and novel site-specific small-molecule reactivators restoring wild-type activities could be used to treat polymorphic ALDH2 related diseases [76]. 

Less toxic acetic acid is activated in the body to acetylCoA by ATP-dependent acetylCoA synthetase by the following reaction:



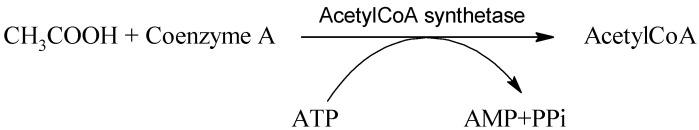

(5)


The acetylCoA formed may be used in the tricarboxylic cycle or diverted to de novo fatty acid synthesis. AcetylCoA availability modulates many substrate-level protein acetylation modifications [77].

### 3.2. Other Minor Metabolites of Ethanol

Small portions of ethanol are metabolized via the nonoxidative pathway of ester formation. This minor nonoxidative pathway may be activated after inhibition of major oxidative pathways, e.g., through the inhibition of ADH. This happens mainly in the liver and pancreas [78]. In addition, some of these processes have been implicated as mediators of ethanol-induced organ damage [79].

Ethanol is a substrate in the esterification reactions with the following:

A. Fatty acids producing fatty acids ethyl esters (FAEEs) by the action of diverse fatty acid ethyl ester synthases that include a carboxyl ester lipase, also known as a bile salt-activated lipase [80], which is the fate of approximately 0.1% of ingested ethanol. FAEEs persist for at least 24 h even after the ethanol has been eliminated and is no longer detectable. The predominant FAEEs species are ethyl palmitate (E16:0) and ethyl oleate (E18:1), and albumin is their main carrier in blood plasma [79]. FAEEs can also be found in the core of lipoproteins along with other neutral lipids [81].
B. Phospholipids producing phosphatidyl ethanol (PEt) in the process of transphosphatidylation due to the action of membrane-associated phospholipase D2, which involves an exchange of ethanol for the choline of pre-existing phosphatidylcholine [82,83]. Minor metabolites may also serve as promising alcohol consumption biomarkers. Contrary to direct alcohol biomarkers ethyl glucuronide and ethyl sulfate, which can be detected several days after alcohol consumption, phosphatidyl ethanol incorporated into the red blood cell membrane has a half-life up to 28 days after consumption. [84]. High levels of PEt after alcohol intake in the lungs, stomach, spleen, small intestine, large intestine, kidney, liver, and heart have been observed. However, PEt was detected in thymus only after high-dose usage and in skeletal muscle only after high-dose chronic usage. On the contrary, it was not detected in the pancreas and testis at all [85].

In a specific area of the brain, the posterior ventral tegmental area, the production of the alkaloid salsolinol (1-methyl-6,7-dihydroxy-1,2,3,4-tetrahydroisoquinoline), the product of condensation between dopamine and acetaldehyde created spontaneously (Pictet–Spengler reaction) or by the action of salsolinol synthase can be detected. Salsolinol is responsible for dopamine release into the ipsilateral nucleus accumbens shell via μ-opioid receptor stimulation. Neurons from locations associated with the synthesis or action of salsolinol are key components of the brain motivation circuit activated by addictive drugs. Salsolinol can be metabolized by N-methyltransferase to N-methyl(R)salsolinol and subsequently via amine oxidase to 1,2-dimethyl-6,7-dihydroisoquinolinium ion. Both salsolinol and N-methyl-salsolinol are found in the cerebrospinal fluid of patients with Parkinson’s disease [86,87,88].

## 4. Toxicity of Ethanol

### 4.1. General Effects

Various behavioral and metabolic effects occur after the ingestion of alcoholic beverages. Blood alcohol levels around 12 mM are associated with anxiolytic and euphoric effects. An increase in ethanol concentration is related to slowed response times, motor and cognitive impairment, and increased sedation. Above 50 mM, this level of sedation and respiratory depression can lead to coma or death [89]. Tolerance to acute manifestations may develop, and adapted humans may survive eight-fold higher concentrations compared to ethanol-naive persons [90]. Ethanol itself but also some compounds resulting from its metabolization, such as acetaldehyde, NADH, acetylCoA, FAEEs, and PEt, cause general manifestations related to toxicity. 

Ethanol crosses the blood–brain barrier and has both stimulating and sedative effects on the central nervous system. Alcohol-induced sedative effects reflect a general decrease in cerebral cortex activity related to the reduced glucose metabolism in the entire brain [91]. Ethanol nonspecifically and transiently directly or indirectly interacts with many molecules transmitting signals, such as receptors, neurotransmitters, or neurohormones. A well-described direct interaction is between ethanol and proteins with a receptor role. Cys-loop ligand-gated ion channels (LGICs) are a superfamily comprising nicotinic acetylcholine, GABA_A_, and glycine receptors, for which they are described as specific sites sensitive to ethanol [92]. Abrahao et al. [93] summarize brain molecules with direct and indirect interaction with ethanol. Among the proteins with direct interactions are mainly membrane channels and receptors, such as N-terminal and transmembrane 3 domains of NMDA receptors, α-subunit of high-conductance Ca^2+^-activated K^+^ channel (BK channel), and 43-amino-acid C-terminal region of G-protein-coupled inwardly rectifying K^+^ channel (GIRK). Ethanol can also directly interact with intracellular protein kinase C and adenylate cyclase. The indirect action of ethanol with an unknown ethanol-binding site involves a much wider range of molecules. It affects the small-conductance Ca^2+^-dependent K+ (SK channel), hyperpolarization-activated and cyclic nucleotide-gated channel (HCN channel), dopamine system, opiate, ionotropic and metabotropic glutamate, taurine, GABA, cholinergic, serotoninergic, corticotropin-releasing factor, and NO signaling. The myriad of the effects on the mentioned molecules is the cause of ethanol-related chronic and acute symptoms, including behavioral changes, withdrawal syndrome, alcohol dependency, or epilepsy [94].

In addition to ethanol, also the ethanol ester PEt may have a direct effect on the central neural system. PEt competes with phosphatidic acid and phosphatidylinositol bisphosphate and antagonizes K^+^ channel ion flux, leading to a change in membrane polarization and nerve excitability. Among the ion channels inhibited are TREK-1, TRAAK, and IRK potassium channels [95].

Hangover severity significantly correlates with markers of inflammatory response interleukin 6, tumor necrosis factor-alpha (TNF-α), and C-reactive protein. Ethanol alone is associated with elevated interleukin 6, suggesting a direct proinflammatory action of ethanol [96], and it enhances lipopolysaccharide-stimulated inflammatory response [97]. Ethanol also activates the complement system (C3, C4), which interacts with Kupffer cells resulting in the production of TNF-α, interleukin 6, and interleukin 10. TNF-α, palmitic acid, downregulation of proteasome functions, and interleukin 17 cause hepatocytes to produce chemokines for neutrophil recruitment [98]. After the first step of oxidation of ethanol, acetaldehyde is elevated. Acetaldehyde is a transient toxic intermediate. Although it should be immediately oxidized to acetic acid, it can accumulate and is responsible for many symptoms related to a hangover. Acetaldehyde markedly increases TNF-α, interleukin 8, and interleukin 1β, increases lipid peroxidation damage, and decreases catalase activity in hepG2 cells [99]. Furthermore, the reactive carbonyl group of the acetaldehyde can bind compounds with amino, hydroxyl, and sulfhydryl groups [100], which gives acetaldehyde the potential to modify the structure and function of many macromolecules, such as proteins, saccharides, and nucleic acids [98].

Of particular importance is the ethanol-induced oxidative stress, which contributes to various pathological conditions, such as inflammation or carcinogenesis, and causes dysfunction of barriers securing the homeostasis, including the intestinal [101] or blood–brain barrier [102]. For example, the treatment of gastric epithelial cells with 10% ethanol enhanced ROS production from mitochondria, and exposure to 15% ethanol caused immediate cell death within 1 h [103]. The development of oxidative stress takes place in several main pathways. Acetaldehyde mediates an increase in nitrogen and oxygen reactive species via induction of nitric oxide synthase, NADPH oxidase, and xanthine oxidase on post-transcriptional level [104]. Acetaldehyde-induced reactive oxygen species have many pathogenetic effects and cause mitochondrial fragmentation due to induced phosphorylation and translocation of dynamin-related protein-1 (Drp1) into mitochondria and elevation of Ca^2+^ and activation of Ca^2+^-calmodulin-dependent protein kinase II (CaMKII) [105]. Another important source of reactive oxygen species after alcohol consumption is CYP2E1, which is capable of producing ethoxy radical, hydroxyethyl radical, acetyl radical, singlet radical, superoxide radical, hydrogen peroxide, hydroxyl radical, alkoxyl radical, and peroxyl radical [106]. Redox dysbalance is supported by the depletion of NADPH, which is necessary for the regeneration of glutathione, an essential antioxidant molecule and cosubstrate for antioxidant enzymes [107]. 

Damage to macromolecules such as lipids, DNA, and proteins is a common toxic effect of alcohol consumption associated with cell viability, proliferation, and tumorigenesis, which is usually mediated via covalent modification. Excessive or prolonged alcohol drinking promotes the formation and accumulation of advanced glycation end products [108]. Oxidative stress induced by reactive species causes lipoperoxidation and increases malondialdehyde (MDA), hydroxyethyl radical (HER), and hydroxynonenal (HNE) [109]. These compounds react with membrane lipids, such as phosphatidylethanolamine or phosphatidylserine, to produce phospholipids with modified solubility, leading to changes in the biophysical properties of the membrane. In addition, lipoperoxidation products non-enzymatically react with proteins through Michael additions and Schiff base formation and generate lipid and DNA adducts. [110]. HNE has been linked to site-specific mutations of the *p53* gene [111]. Acetaldehyde reacts with a myriad of proteins forming unstable or stable adducts. Schiff bases are unstable and readily reversible. Stable adducts are irreversible, forming fluorescent products. At least two amino groups are thought to be involved in a stable fluorescent adduct responsible for forming inter- or intra-molecular cross-links, and the generation of a crotonaldehyde Schiff base is essential for this process [112]. Acetaldehyde with malondialdehyde forms hybrid protein adducts (MAA) with immunogenic properties. The body produces antibodies against these adducts. The level of IgG antibodies against MAA correlates with the severity of liver damage. Thus, MAAs serve as antigens provoking immunologic reactions associated with ethanol-related liver damage [113]. In the liver, MAA adducts also exhibit profibrogenic properties [114]. Albumin-bound MAAs induce direct cytotoxicity on monocytes [115]. Albumin- and LDL-bound MAAs stimulate endothelial cells and macrophages to increase cytokines expression. LDL-MAA increased the expression of TNF-α in macrophages and MCP-1 in endothelial cells, and albumin-MAA increased the expression of interleukin 6, TNF-α, ICAM-1, and VCAM-1 in endothelial cells and interleukin 6 and TNF-α in macrophages [116]. This proinflammatory response contributes to the symptoms typical of a hangover. In addition to the great potential for altering the structure of macromolecules and stimulating the pathological immune response, acetaldehyde has multiple cytotoxic effects on voltage-dependent Ca^2+^ channels that cause myocardial depression and the release of epinephrine, norepinephrine, histamine, and bradykinin, leading to facial flush and vasodilation [117].

In addition to proteins, acetaldehyde reacts with nucleophiles to generate covalent adducts with nucleic acids, and the disruption of DNA repair mechanisms dramatically influences acetaldehyde tolerance. At the same time, unrepaired DNA adducts propagate as mutations after DNA replication. [110]. Recently, Guidolin et al. [118] identified by DNA adductomic approach 22 DNA adducts in DNA exposed to acetaldehyde, and adduct levels were observed to generally increase with acetaldehyde concentration for all the detected modifications. Out of 22 DNA adducts, 17 were detected in oral DNA samples. 

NADH, the by-product of ethanol metabolization, is also responsible for many well-described acute biochemical effects. The sudden overproduction of NADH in reactions catalyzed by ADH and ALDH is responsible for the severe acute symptoms of intoxication. This process leads to a shift in lactate dehydrogenase reaction and production of lactate. Moreover, NADH surplus decreases the rate of the tricarboxylic cycle, impairs glycolysis, and inhibits gluconeogenesis. Hypoglycemia is related to the activation of stress hormones, which together with overproduction of the final product of ethanol oxidation acetylCoA, which cannot be utilized in an inhibited tricarboxylic cycle, support and amplify ketogenesis. Carnitine plays an important role in processing a pool of newly formed acetylCoA following alcohol consumption and reduces withdrawal symptoms [119]. Acidosis caused by the lactate and ketone bodies’ overproduction is the underlying cause of severe metabolic acidosis related to alcohol intake. Metabolic acidosis has common consequences: vomiting leading to dehydration, hypotension, and loss of chlorides, and potassium disturbances. A decrease in hemoglobin saturation with the oxygen due to acidosis and depression of the brain’s breathing center worsen NADH reoxidation. Emergency care focuses mainly on coping and improving these mentioned acute metabolic and neurological symptoms in ethanol-intoxicated persons. Ethanol itself is an osmotically active compound causing the change in osmolarity. There are many equations published to calculate the osmolal gap caused by ethanol useful in acute medicine [120]. Changes in osmolality support the water and ion imbalance in cells. There is an inverse relationship between the osmolal and anion gap [121]. Calculation of the osmolal gap may help in the diagnosis and treatment of disorders of the internal environment.

In heavy drinkers, alcohol consumption is related to nutrients and essential compounds disturbances caused by insufficient intake or higher depletion. In case of deficiency of essential compounds, clinical symptoms occur. Among those well described are vitamin deficiencies, which may support a dramatic worsening of ethanol-related pathological conditions. A wide range of presentations related to vitamin deficiencies in alcohol consumers may be visible in the oral cavity, such as inflammatory changes, hemorrhages, ulcers, precancerosis, sensory presentations, or salivary gland problems [122]. Alcohol consumption decreases the transport of thiamine (vitamin B1) across the intestinal mucosa, decreases the storage of thiamine in the liver, and impairs the activation by inhibiting thiamine phosphorylation to thiamine diphosphate [123]. Thiamine is vital in energetic metabolism, and thiamine deficiency may associate with cardiovascular diseases in alcohol consumers [124]. Ethanol induces the impairment of phosphorylation of vitamin B6 and increases the rate of its degradation [125]. Folate deficiency is also common. In patients with alcohol use disorder, the deficiency is probably a combination of the following factors: inhibited transcription of the reduced folate carrier in the intestine [126], impaired hydrolysis of polyglutamate forms of folate to absorbable monoglutamate forms by intestinal γ-glutamate carboxypeptidase [127], decreased ability to retain folate in the liver [128], and increased urinary excretion [129]. Folate is closely related to the metabolism of amino acid methionine and the synthesis of nucleotides. Speculation exists about the extensive downregulation of B vitamins as the potential mechanism of cardiovascular disease [130]. There is also some tissue-specific information about ethanol-induced damage.

### 4.2. Mouth

Ethanol causes the denaturation of proteins via dehydration. At a 60–80% concentration, ethanol is widely used as disinfection with a potent bactericidal and virucidal effect [131]. Similarly, this “drying effect” is on mucous surfaces of the mouth and upper part of the gastrointestinal tract after ingesting concentrated alcoholic beverages. About 30 min after alcohol ingestion, higher ethanol concentrations occur in saliva and salivary glands than in blood [122]. In short-term experiments, the direct local toxic action of the alcohol leading to damage of the mucous membrane proportional to the degree of alcohol concentration was proven [132]. Ethanol increases the permeability of oral mucosa, can decompose the lipid composition of the outer epithelial membrane, and increases the sensitivity to other dangerous compounds, e.g., carcinogens from smoking. The recent history of alcohol drinking in young adults was related to significant differences in the cell viability, DNA fragmentation index, and mitochondrial function of oral mucosa cells [133]. In addition to ethanol’s direct toxic effects, enzymes involved in ethanol metabolization increase the possible cell damage in the oral cavity. The origin of salivary alcohol dehydrogenase activity comes from the oral mucosa, salivary glands, and microbiota. Ethanol is oxidized to acetaldehyde, but in human saliva, ALDH3 isoform of aldehyde dehydrogenase is found [67], for which acetaldehyde is not the favorite substrate under basal conditions. Thus, the oral cavity is exposed to high concentrations of acetaldehyde for a long time. Long-term studies show dyskeratosis, keratosis, thickening of the basal layer, and higher mitotic activity [132]. Chronic exposure to ethanol causes cancer-like cytologic changes in the oral mucosa (increased pyknosis, karyorrhexis, karyolysis, nucleus/cytoplasm ratio, and micronuclei) without correlation with hepatobiliary injury [134], and the consumption of alcohol lasting more than 35 years is the significant risk factor of the oral cavity and pharynx cancer [135]. Of 60 people who died in relation to chronic heavy drinking, 10% had epithelial hyperplasia and 90% had epithelial atrophy with lymphocyte-macrophage infiltration in basal oral mucosa [136]. Recently, Ivoš et al. [122] reviewed the effects of ethanol on oral health, among which were damage of mucosal and glandular tissues, reduction in immune functions, an increased rate of various inflammatory oral diseases, peripheral neuropathy connected to sialoadenosis, reduction of saliva excretion, precancerous lesions, or cancer. The author also discussed secondary effects of alcohol abuse such as poor dental hygiene causing a higher incidence of tooth decay, dental caries, periodontal diseases, and permanent tooth loss or nutritional deficiencies with oral presentations. 

### 4.3. Gastrointestinal Tract

Gastrointestinal tract epithelium is one of the fastest renewing tissues, a vital barrier, an immune organ, and ethanol’s first-pass metabolism site. Consequently, ethanol has pathological effects on intestinal function due to loss of barrier integrity, causing local and systemic inflammation and microbial dysbiosis [101].

In the stomach, ethanol may cause injury via distinct possible mechanisms. Cyclic AMP is an important regulator of gastric acid secretion and control of the integrity of the cells. Ethanol decreases cyclic AMP levels in gastric mucosa by decreasing gastric mucosal adenylyl cyclase activity [137]. Ethanol also affects the synthesis, transport, and processing of gastric mucus glycoprotein, leading to a dramatic decrease in the delivery of mucus glycoprotein to the gastric epithelial surface [138]. The oxidation of cellular lipids and proteins after alcohol administration is another cause of disruption of the gastric mucosal barrier, supporting the extravasation of leukocytes. After alcohol administration, significantly elevated malondialdehyde and decreased levels of reduced glutathione in gastric tissue were found. This damage may be attenuated by caffeic acid treatment [139].

In the intestine, isoenzyme ALDH2 is highly expressed. The accumulation of toxic acetaldehyde caused by the mutation of ALDH2 in the intestine disrupts the barrier function, specifically intestinal epithelial tight junctions, adherent junctions, and liver damage [140]. ALDH2-deficient humans who habitually consume alcohol have a higher cancer rate than humans with functional ALDH2 [141]. The relation to esophageal cancer has been well described [75].

Excessive alcohol intake induces morphological changes of the villus surface and crypt deformation similar to intestinal bowel diseases. Moreover, ethanol stimulates the proliferation of the small intestinal epithelial cells and intestinal stem cells in intestinal crypts via increased expression of *cyclin D1, Ki67* which are proliferation markers and Wnt target genes essential in the self-renewal of small intestinal stem cells and the generation of Paneth cells located nearby the stem cells [142]. 

Some lipid molecules may have protective effects against alcohol-induced injury, e.g., long-chain fatty acids (LCFA), tributyrin, or polyunsaturated fatty acids (PUFAs), preventing intestinal hyperpermeability on the transcriptome level and modulating the gut microbiome. A study on ethanol-induced intestinal transcriptome changes shows the downregulation of genes in pathways related to the hormones, hemoglobin, and innate immunity and upregulated genes related to tuft cell markers, lectin recognition, fibroblast growth factor, and inflammation. These changes differ significantly from those detected in the presence of PUFA. Ethanol also reduces the expression of CD73 (ecto-5′ nucleotidase), which is responsible for the conversion of ATP to adenosine, which is an important signal in the anti-inflammatory pathway [143]. 

Excessive alcohol consumption is a risk factor associated with cancers, including gastrointestinal cancers. Regarding the colorectum, studies show the protective effect of low-to-moderate drinking on proliferation and tumorigenesis in the colorectum, with a lower cell proliferation index, lower proliferative zone, decreased cell differentiation, and apoptosis, mainly on older subjects. Diet, genetics, other carcinogens intake, or various pathologies may be modulating factors for the carcinogenic potential of ethanol [144].

### 4.4. Musculoskeletal System

In bone marrow and reticulocytes, ethanol inhibits hemoglobin synthesis. Inhibition is dose-dependent, and the synthesis of the globin chain is more affected [145]. Chronic alcohol intake also directly affects bone health, decreases bone mass, and increases the risk of hip fracture. Oxidative stress associated with acetaldehyde production impairs osteoblastogenesis. Single nucleotide polymorphism rs671 in the *ALDH2* gene is associated with osteoporosis in the affected individuals. ALDH inhibitors may have side effects on bones. Organic compound Alda-1 is the first activator of ALDH2, which via ALDH2 increases the expression of bone morphogenic protein 2 (BMP2) and induces osteoblast differentiation. Thus, Alda-1 like molecules could serve as a potential osteoanabolic drug [146].

A higher concentration of MAAs associated with tolerance loss was found in synovial tissues in patients with rheumatoid arthritis. Circulating antibodies against MAA correlated with seropositivity for rheumatoid factor and anti-citrullinated protein antibodies. MAA formation may result in autoimmune responses typical for patients with rheumatoid arthritis. Alcohol consumption may also support autoimmune diseases [147].

### 4.5. Pancreas

Although alcohol is a well-known risk factor for developing pancreatitis, the exact mechanism is not well understood. Novel studies show possible pancreas-specific effects of FAEEs, which are products of the nonoxidative metabolism of ethanol, probably exhibiting direct toxicity on the pancreas. It is established that FAEEs may bind to intracellular membranes, causing a disordering effect [148] and increasing lysosomal fragility [149]. Werner et al. [78] reported ethanol-induced pancreatic injury with edema, intrapancreatic trypsinogen activation, and vacuoles formation in acinar cells associated with FAEEs. Trypsin activation and acinar cell injury were also observed in another study. In addition, FAEEs induced the redistribution of cathepsin B from lysosomes to zymogens after a 30 min treatment. One hour after the treatment, caspase-3 and annexin-V apoptotic markers were increased and continued to rise even after two hours. The release of interleukin 6, interleukin 8, and TNF-α cytokines was also detected in a time-dependent manner in human pancreatic acinar tissues in an ex vivo model [150]. Circulating FAEEs are 20–50-fold increased during alcoholic pancreatitis, but not in the serum of intoxicated persons, and they could serve as a biomarker of alcoholic pancreatitis. The FAEEs increase is independent of the actual ethanol level. Authors have speculated about the probable origin of FAEEs from visceral release [151]. The use of carnitine may decrease alcohol-induced FAEE production [119].

### 4.6. Cardiovascular System

Ethanol is a well-described risk factor for many cardiovascular diseases related to structural and functional abnormalities. Increased alcohol drinking is positively associated with increased blood pressure levels, but only in men [152]. Furthermore, ethanol has significant arrhythmogenic potential. Atrial fibrillation is the most common alcohol-related arrhythmia, even in low/moderate alcohol intake. On the other hand, low/moderate consumption protects against severe ventricular arrhythmias and cardiac arrest [153]. Beneficial effects of low/moderate drinking of alcohol on cardiovascular diseases could be mediated via various molecules related to basic nutrient metabolism (higher HDL cholesterol, higher adiponectin, high insulin sensitivity), to coagulation (reduction of endothelial dysfunction, influencing coagulation factors, fibrinolysis), inflammation, and level of atrial natriuretic peptide [154,155]. High alcohol consumption-associated arrhythmias are related to myocardial inflammation, leading to increased troponin concentration in blood [156]. Alcoholic cardiomyopathy associated with interstitial fibrosis, dilatation of ventricles, reduced cardiac output, high risk of arrhythmias, stroke, or hypertension is well-described [157,158,159] with 4-year mortality close to 50% [155]. 

The mechanism of myocyte damage is related to the reduction of mitochondrial proteosynthesis, mitochondria damage, oxidative stress, apoptosis, modification of contractile proteins actin and myosin, lysosomal proteases-mediated loss of individual myocardial proteins, and disturbances in calcium metabolism [154,156,160]. Although alcoholic cardiomyopathy should be independent of nutrition and vitamins [161], thiamine supplementation can improve the ejection fraction in patients with alcoholic cardiomyopathy [123]. Alcohol-induced FAEEs bound to the mitochondrial membrane in myocytes cause mitochondrial dysfunction by impaired coupling of the respiratory chain with oxidative phosphorylation [162]. Acetaldehyde probably plays a significant role in direct or indirect toxicity on myocytes. It is clear that the toxicity lies in the mutated allele ALDH2*2 [163]. East Asians may confer subclinical damage of cardiac function also under moderate alcohol intake conditions [164]. Flushing related to acetaldehyde action after alcohol consumption can also signify a higher risk of ethanol-related heart complications [165]. According to a novel metabolomic study, patients with alcoholic cardiomyopathy show changes in many metabolites. These were related to the biosynthesis of the unsaturated fatty acids pathway (downregulated levels of adrenic acid, docosapentaenoic acid, docosahexaenoic acid, and arachidonic acid; upregulated levels of alpha-linolenic acid, stearic acid, arachidic acid, and palmitic acid), vitamin digestion and absorption pathways (downregulated levels of thiamine monophosphate, panthotenic acid, nicotinamide, and riboflavin), oxidative phosphorylation pathway, pentose phosphate pathway, purine and pyrimidine metabolic pathways (donwregulated xanthine, thymine, cytidine, adenylosuccinic acid, ribulose-5-phosphate, sedoheptulose-7-phosphate, uridine diphosphate), phosphatidylcholine, lysophosphatidylcholine, and fatty acid esters of hydroxy fatty acids [130]. Similar studies could bring a novel and more complex view on ethanol-induced heart damage. 

### 4.7. Brain

In the central nervous system, the region-specific metabolism of ethanol is present. For most cells, the dominant ethanol-oxidizing enzymes are catalase (60%, Km = 12 mM) and CYP2E1 (20%, Km 8–10 mM) [166]. The presence of alcohol dehydrogenase is limited on low expressed ADH1B and ADH1C in the cerebellum (Table 1). The mitochondrial isoform of ALDH2 oxidizes acetaldehyde to acetate [58]. Since ethanol processing in the central nervous system uses catalase or CYP2E1 in the first step, it is more associated with oxidative stress in the CNS than in other localities. In the brain, the toxic effects of ethanol and acetaldehyde described earlier predominate. Concentrations of acetaldehyde in the blood and brain do not correspond, as the blood–brain barrier is not permeable for acetaldehyde [167]. In astrocytes, acetaldehyde is a more potent toxin than ethanol, and both modulate the production of proinflammatory interleukin 6 and TNF-α [168]. The overproduction of NADH in the brain is lower than in other cells, mainly using ADH as an ethanol-oxidizing system. Acetate produced from acetaldehyde can be converted to neurotransmitter acetylcholine or used in the tricarboxylic cycle in the form of acetylCoA. AcetylCoA can compensate for energy deficit caused by ethanol-induced decreased glucose uptake. At the same time, glucose is also essential for the pentose phosphate pathway as an NADPH source. Inherited tolerance to the hypnotic effect of ethanol in rats is related to the better utilization of acetate for acetylCoA and acetylcholine synthesis [169]. In the central nervous system, there are also interesting co-addictions involving ethanol, for example, the co-addiction of ethanol and nicotine [166]. Minor metabolites of ethanol as PEt or salsolinol may become more important in understanding the ethanol-induced effects in the brain in the future. 

### 4.8. Liver

Concerning ethanol toxicity, liver damage is one of the best studied and includes a spectrum of disorders, such as fibrosis, steatosis, cirrhosis, alcoholic hepatitis, and hepatocellular carcinoma. Toxicity in the liver is related to the overproduction of NADH, acetaldehyde, acetylCoA, but also oxidative stress, and many effects overlap. 

Increased NADH inhibits beta-oxidation and stimulates lipogenesis. Alcohol-induced inhibition of beta-oxidation is also mediated by the inhibition of PPAR-α (peroxisome proliferator-activated receptor alpha), which is downregulated directly via acetaldehyde- or CYP2E1-related oxidative stress, zinc deficiency, adenosine, and adipokine signaling [98]. In addition to the lipogenic effect of NADH, it also inhibits the deacetylase activity of SIRT1 (sirtuin-1) [170], leading to the activation of the p53 protein with a pro-apoptotic effect [171].

AcetylCoA serves as a precursor for fatty acid synthesis, which involves alcohol-induced transcription factors SREBPs (sterol regulatory element binding transcription factors) [172], which regulates the expression of genes involved in lipid synthesis [173]. The higher availability of acetylCoA originating from ethanol oxidation increases the acetylation of proinflammatory gene histones with subsequent enhancement of response in liver macrophages supporting the development of acute alcoholic hepatitis [174].

In the liver, the minor CYP2E1 pathway is a massive generator of reactive oxygen species causing lipid peroxidation. Chronic alcohol drinking induces CYP2E1 activity and increases oxidative stress, resulting in depleting glutathione and S-adenosylmethionine (SAM) [98]. SAM is a precursor for polyamine synthesis necessary for cell viability and proliferation and is the principal methyl donor required for the methylation of nucleic acids, biogenic amines, phospholipids, histones, and other proteins. In the liver, SAM is an important precursor for glutathione synthesis. SAM depletion can be explained via ethanol-induced inactivation of methionine adenosyltransferase, excessive hepatic consumption of SAM, or impaired betaine-mediated homocysteine methylation, leading to inhibition of vitamin B12 and folate-dependent methionine synthesis [175]. Thus, lower SAM concentrations lead to increased levels of homocysteine. Excessive homocysteine induces the accumulation of misfolded proteins, resulting in endoplasmic reticulum (ER) stress [176]. ER stress activates interferon regulatory factor 3 (IRF3), which modulates the inflammatory functions in liver macrophages [177] and is required for the mitochondrial pathway of hepatocyte apoptosis [178]. ER stress is also related to changes in membrane cholesterol content and Insig (significant integrator of nutrient and hormonal signals) level [179], which lead to the upregulation of SREBPs [172,180]. SREBPs can also be upregulated directly via acetaldehyde, or indirectly through adenosine, endocannabinoids, sirtuin1, adiponectin, STAT3 (signal transducer and activator of transcription 3), and lipopolysaccharide signaling [98]. 

Acetaldehyde can form various adducts of DNA and proteins, especially in the liver. High anti-MAA antibody titers have been associated with the severity of liver damage [114]. Alcohol consumption can also indirectly modify many other factors, including hypoxia-inducible factor-1 (HIF-1), C3, C1qa, PKCε, and inducible nitric oxide synthase, which all contribute to the development of steatosis [98]. Injured hepatocytes produce damage-associated molecular patterns (DAMPs), leading to changes in recognition receptors and the production of proinflammatory cytokines [98]. TNF-α is required for ethanol-induced liver damage [99].

The gut microbiome also may contribute to ethanol-related liver damage via a change in gut permeability. For example, bacterial metabolites and cell components, such as lipopolysaccharides, can be translocated from the gut through the intestinal barrier into the portal vein and transported to the liver, where they interact with liver cells and lead to inflammation and steatosis [14].

The epigenetic regulatory mechanism also may be applied to ethanol metabolization. For example, key alcohol-metabolizing enzymes are repressed via newly described regulation by miRNA in patients with alcoholic hepatitis [181].

### 4.9. Lung

Mammalian lungs can metabolize ingested ethanol by ADH but also microsomal CYP2E1 and peroxisomal catalase [48]. Alcohol intake is related to a higher incidence of lung inflammatory diseases with augmented production of proinflammatory cytokines, particularly interferon γ (IFNγ) and interleukin 1β in response to lipopolysaccharide and lipoteichoic acid [182]. Alcohol-induced susceptibility to infections contributes to the increased morbidity and mortality of alcohol users [183]. Several underlying mechanisms may be proposed. Ethanol directly causes the desensitization of cilia in the upper airway, which decreases cilia motility and pathogen clearance. Chronic alcohol ingestion induces oxidative stress within the microenvironment of the alveolar space, which impairs the capacity of alveolar macrophages to phagocytose and clear bacteria [184]. In addition, protein adducts play a role in lung damage. In the lungs, MAA bind to the surfactant protein D (SPD). The major receptor for these adducts is scavenger receptor A1, among others, which is present in bronchial epithelial cells and macrophages. SPD-MAA decreases lung cellularity, increases the influx of neutrophils in the lungs, increases keratinocyte chemoattractant (KC), increases the expression of scavenger receptor A1, and also increases peribronchiolar inflammation [185]. 

## 5. Conclusions

Alcohol is one of the commonly used drugs. Ethanol, together with its derivatives and metabolites, exhibits diverse direct and indirect toxic effects, which are responsible for damage to many organs. This toxicity depends on many factors, such as dose, gender, associated comorbidities, or genetic predisposition. There are still many gaps in understanding for all the interrelations between tissue damage and the metabolization of alcohol. 

## Figures and Tables

**Table 1:** Classes of ADH and tissue/cell ADH expression based on information mainly from Human Protein Atlas.

Class	ADH Subunit	Tissue Expression	Cell Compartment	Substrates
I	ADH1A active in fetal life low in adults	MPES: liver LPES: duodenum, small intestine [30]	⮚ PM ⮚ cytosol [31]	⮚ ethanol ⮚ retinol ⮚ other aliphatic alcohols ⮚ hydroxysteroids ⮚ lipid peroxidation products [27,28,29]
	ADH1B major role in ethanol oxidation	HPES: liver MPES: duodenum, small intestine LPES: cerebellum, oral mucosa, esophagus, stomach, rectum, gallbladder, kidney, urinary bladder, vagina, breast, heart muscle, appendix, tonsils [32]	⮚ PM ⮚ cytosol [33]	
	ADH1C major role in ethanol oxidation	HPES: liver MPES: stomach, duodenum, small intestine, rectum, gallbladder, kidney, urinary bladder, epididymis, seminal vesicle, breast, appendix LPES: cerebellum, basal ganglia, hippocampus, colon, testis, vagina, fallopian tube, heart muscle, skeletal muscle, adipose tissue, spleen, lymph node, tonsil, bone marrow [34]	⮚ PM ⮚ cytosol [35]	
II	ADH4	HPES: liver MPES: duodenum LPES: small intestine [36]	⮚ nucleoplasm ⮚ cytosol [37]	⮚ retinol ⮚ long chain omega-hydroxy fatty acids ⮚ benzoquinones [38]
III	ADH5 ineffective in ethanol oxidation	HPES: liver, kidney, testis, epididymis, smooth muscle MPES: thyroid gland, adrenal gland, nasopharynx, bronchus, lung, oral mucosa, salivary gland, esophagus, stomach, duodenum, small intestine, colon, rectum, gallbladder, pancreas, urinary bladder, seminal vesicle, prostate, vagina, ovary, endometrium, cervix, heart muscle, skin, appendix LPES: cerebral cortex, hippocampus, parathyroid gland, fallopian tube, placenta, breast, tonsil [39]	⮚ general cytoplasmic expression [40]	⮚ long-chain primary alcohols ⮚ S-hydroxymethyl-glutathione ⮚ formaldehyde [40]
IV	ADH6 contains steroid hormone receptor binding site [41]	MPES: liver, gallbladder, duodenum, small intestine LPES: stomach, rectum, kidney [42]	⮚ N/A, cytosol? [43]	
V	ADH7 ineffective in ethanol oxidation	HPES: nasopharynx, bronchus, oral mucosa, esophagus MPES: stomach, tonsil LPES: colon [44]	⮚ PM ⮚ cytosol [45]	⮚ retinol derivatives, dietary alcohols (2-hexenol, juniperic acid) [46]

Legend: HPES—high protein expression score, MPES—medium protein expression score, LPES—low protein expression score, PM—plasma membrane.
